# Genome-wide analysis identifies rare copy number variations associated with inflammatory bowel disease

**DOI:** 10.1371/journal.pone.0217846

**Published:** 2019-06-11

**Authors:** Svetlana Frenkel, Charles N. Bernstein, Michael Sargent, Qin Kuang, Wenxin Jiang, John Wei, Bhooma Thiruvahindrapuram, Elizabeth Spriggs, Stephen W. Scherer, Pingzhao Hu

**Affiliations:** 1 Department of Biochemistry and Medical Genetics and The George and Fay Yee Centre for Healthcare Innovation, University of Manitoba, Winnipeg, Manitoba, Canada; 2 Department of Internal Medicine and the University of Manitoba IBD Clinical and Research Centre, University of Manitoba, Winnipeg, Manitoba, Canada; 3 Division of Biostatistics, Dalla Lana School of Public Health, University of Toronto, Toronto, Ontario, Canada; 4 The Centre for Applied Genomics, Genetics and Genome Biology, the Hospital for Sick Children, Toronto, Ontario, Canada; 5 Department of Biochemistry and Medical Genetics, the University of Manitoba and Molecular Diagnostic Laboratory, Diagnostic Services of Manitoba, Winnipeg, Manitoba, Canada; 6 Department of Molecular Genetics, University of Toronto, Toronto, Ontario, Canada; 7 Department of Electrical and Computer Engineering, University of Manitoba, Winnipeg, Manitoba, Canada; University of Toronto, CANADA

## Abstract

**Background:**

Inflammatory bowel disease (IBD) is an idiopathic, chronic disorder of unclear etiology with an underlying genetic predisposition. Recent genome-wide association studies have identified more than 200 IBD susceptibility loci, but the causes of IBD remain poorly defined. We hypothesized that rare (<0.1% population frequency) gene copy number variations (CNVs) could play an important mechanism for risk of IBD. We aimed to examine changes in DNA copy number in a population-based cohort of patients with IBD and search for novel genetic risk factors for IBD.

**Methods:**

DNA samples from 243 individuals with IBD from the Manitoba IBD Cohort Study and 2988 healthy controls were analyzed using genome-wide SNP microarray technology. Three CNV calling algorithms were applied to maximize sensitivity and specificity of CNV detection. We identified IBD-associated genes affected by rare CNV from comparing the number of overlapping CNVs in IBD samples with the number of overlapping CNVs in controls for each gene.

**Results:**

4,402 CNVs detected by two or three algorithms intersected 7,061 genes, in at least one analyzed sample. Four genes (e.g. *DUSP22* and *IP6K3*) intersected by rare deletions and fourteen genes (e.g. *SLC25A10*, *PSPN*, *GTF2F1*) intersected by rare duplications demonstrated significant association with IBD (FDR-adjusted p-value < 0.01). Of these, ten genes were functionally related to immune response and intracellular signalling pathways. Some of these genes were also identified in other IBD related genome-wide association studies. These suggested that the identified genes may play a role in the risk of IBD.

**Conclusion:**

Our results revealed new genomic loci associated with IBD, which suggested the role of rare CNVs in IBD risk.

## Introduction

Inflammatory bowel disease (IBD) is a chronic, progressive and often disabling inflammatory disorder of the gastrointestinal tract associated with dysregulation in both the intestinal immune system and the intestinal microbiota. IBD affects more than 1.5 million people in North America and about 2.5 millions of Europeans with a rising incidence in developing countries.[1,2] Crohn’s disease (CD) and ulcerative colitis (UC) are the two main forms of IBD, both of which are characterised by variations in age of onset, severity of symptoms, disease phenotype, as well as response to treatments.

Numerous genome-wide association studies (GWAS) have identified more than 200 IBD risk loci.[3,4] Many of the candidate genes from the studies linked to IBD are involved in activation of T-, B-, and NK-cells, response to molecules of bacterial origin, JAK-STAT signalling pathway and other processes, which may be linked to the regulation of host response to intestinal microbes.[3,5,6] However, the results of SNP-based GWAS explain only a small fraction of IBD occurrence.[7] Further, investigations are warranted to discover other potential sources of hidden heritability, such as the combined influence of rare genetic variants, large and small structural variations, epigenetic modifications, and other elaborate processes, like gene-gene and gene-environment interactions.

Copy number variations (CNVs) are one of the functionally significant genomic variants that can have critical phenotypic effects caused by gene dosage.[8] By estimation, CNVs cover 4.8–9.5% of the human genome.[9] Currently, more than 552,000 CNV loci are catalogued in the Database of Genomic Variants.[10] CNVs have been associated with numerous diseases and syndromes, including autoimmune [11] and neurodevelopmental disorders.[12,13] There have been some reports of common CNVs involved in IBD. Among them, different studies demonstrated the effect of copy number polymorphism of the *DEFB* genes (8p23.1) on Crohn’s disease predisposition.[14,15] In addition, the CNV upstream of the IRGM gene on 5q33.1 was shown to be associated with CD;[16,17] and three different CNV loci were linked to UC: a duplication at 7p22.1, overlapping RNF216, ZNF815, OCM and CCZ1, a duplication upstream of the KCNK9 gene at 8q24.3 and a deletion at 13q32.1 upstream of ABCC4 and CLDN10.[18] Unlike common CNVs, the contribution of rare CNVs in IBD pathogenesis was not investigated before. Similar to other rare genetic variants, rare CNVs could contribute to the risk of some complex diseases, such as autoimmune disorders.

In the present study, we analyzed CNVs of 243 individuals with CD or UC and 2988 healthy controls using SNP microarray technology. We hypothesized that rare (<0.1% population frequency) CNVs could be susceptibility loci of IBD, which may harbour genes involved in the development of the chronic inflammatory process in the gastrointestinal tract. This study enabled us to detect several rare CNVs overrepresented in IBD patients, which provides new aspects toward understanding disease mechanisms.

## Materials and methods

### Study samples

Individuals were enrolled in The Manitoba IBD Cohort Study–a population-based study of patients with IBD within seven years after diagnosis who were followed prospectively to assess predictors of outcomes.[19] Blood samples were drawn from a total of 269 IBD patients during the period of time from May, 2002 to March, 2004.

We used 2988 healthy control samples with European ancestry from two population-scale studies: KORA (Cooperative Research in the Region of Augsburg)[20] and the COGEND (Collaborative Genetic Study of Nicotine Dependence),[21] which were genotyped using the Illumina Human OMNI 2.5M-Quad microarray. These data were used previously by us [13,22] and others [23] to perform genome-wide case-control CNV comparisons.

### Methods

#### Microarray genotyping and quality control procedures

**Fig 1:**
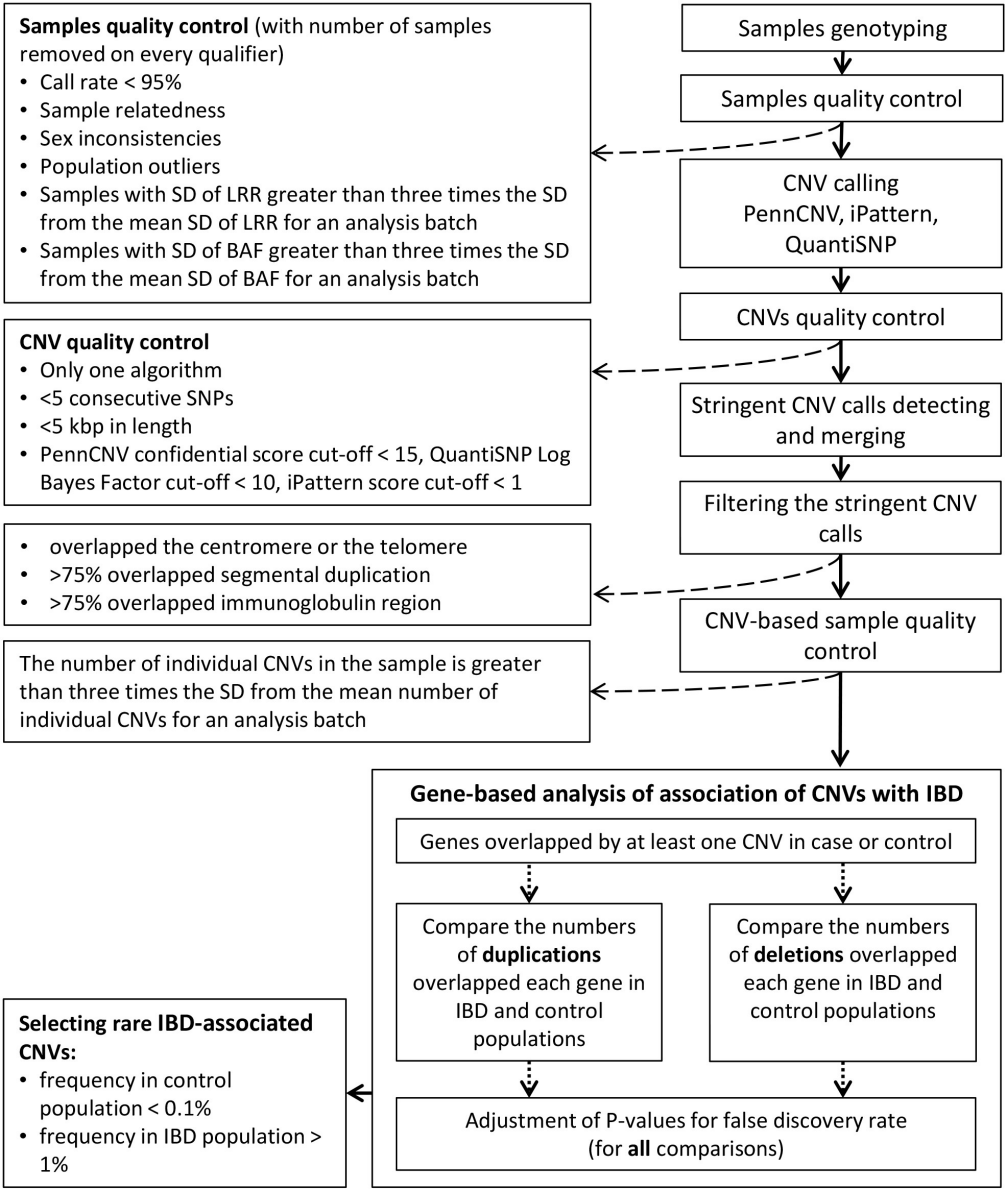
CNV analysis workflow. CNV calling was conducted using variants detected by two or three calling algorithms, which were of sizes greater than 5 kb and spanned at least five array probes. SD: Standard deviation; LRR: Log R ratio; BAF: B allele frequency.

The study was approved by the University of Manitoba Health Research Ethics Board and written informed consents were provided by the participants. DNA of IBD samples was extracted from blood and genotyped using the Illumina Human Omni2.5M-8 microarray (San Diego, CA, USA) at The Centre for Applied Genomics (TCAG) in Toronto using established protocols.[24] IBD and control samples were required to match several quality control criteria (Fig 1): minimal genotype call rate of 95%, the SD (standard deviation) for the LRR (log R ratio) and BAF (B allele frequency) for an individual sample were required to be within the mean ± three times the SD for each of these criteria for an analysis batch.[12,25] Any samples outside this range were removed from further analysis.

#### Population stratification analysis

Population stratification and outlier detection were identified by multidimensional scaling analysis (MDS) as implemented in PLINK.[26], which was performed on an inter-sample distance matrix of IBD patients and the reference populations based on the phase 3 data from 1000 Genomes Project.[27] Only the samples with European ancestry were used for the study. Additionally, the samples sex was estimated based on X chromosome homozygosity rate, and samples relatedness was assessed by calculating the pairwise genotype similarity (identity-by-descent). Four samples with sex inconsistencies and three highly related samples based on identity-by-descent were removed from the analysis.

#### CNVs detecting algorithms

Similarly to the methodology we described before,[12,13,25] we applied three CNV calling algorithms, namely, iPattern,[28] PennCNV,[29] and QuantiSNP,[30] to obtain high-confidence calls from both IBD and control populations. The detected CNVs were first filtered based on their size (no less than five kilobase pairs (kbp), probe content (no less than five consecutive probes) and algorithm-specific quality score (see Fig 1). For maximal sensitivity of CNV detection, we required CNV calling by at least two algorithms. The CNVs detected by two or three algorithms were merged. In the case of position mismatch of the results received from different algorithms, outermost positions were used as stringent CNV positions (i.e., union of the CNVs) as described in Pinto *et al*.[12] Further, we excluded CNVs that: 1) overlapped the centromere (100kbp regions before and after centromeres) or the telomere (100 kb from the ends of the chromosome); 2) had > 70% of its length overlapping a segmental duplication using the entire segmental duplication dataset downloaded from the University of California, Santa Cruz (UCSC) Genome Browser website; 3) had >70% overlap with immunoglobulin region.[9,29]

Additional CNV-based sample quality control was conducted: the individual sample was excluded from the analysis if it provided more CNVs than the average number of CNVs ± three times of the standard deviation of the average number of CNVs for the analysis batch.

#### Genes affected by rare CNVs overrepresented in IBD cohort

The human genes positions (accordingly to GRCh37/hg19 genome assembly) were obtained from the UCSC Genome Browser website. The genes were categorised by the corresponding number of overlapping CNVs in the IBD and control cohorts. All genes, overlapped by any CNV on at least one nucleotide in the tested population, were selected for the further analysis. For each gene, the number of overlapping CNVs in IBD samples was compared with the number of overlapping CNVs in control. The analysis was conducted separately for genes overlapped by deletions and by duplications. For this comparison, we used two-tailed Fisher’s exact test with subsequent correction of p-values for multiple testing using Benjamini-Hochberg procedure.[31] Then, from the genes that passed the significance threshold of adjusted p-value (P_adj_) ≤ 0.01, we selected genes overlapped by rare CNVs. We classified the genes as affected by rare deletions or duplications if they overlapped by corresponding CNVs in less than 0.1% of control samples regardless the CNV frequency in the IBD cohort.

#### Gene set overrepresentation analysis

The overrepresentation analysis of the genes was conducted using ConsensusPathDB.[32] Additional genes encompassed with CNVs associated with IBD with a P_adj_<0.05, were used for the analysis. As a background, we used all genes overlapped by at least one CNV in at least one sample from the IBD or control populations. Four predefined annotation gene set libraries were used: Gene ontology (GO) terms (biological process and molecular function domains), KEGG and Reactome pathways. Only 4 and 5 level GO terms were included.[32] The significance of the gene set overrepresentation was evaluated by Fisher’s exact test. The Benjamini-Hochberg procedure for multiple testing correction was applied within each gene set library we tested. The adjusted p-value threshold of the overrepresentation was relaxed to 0.25 to a better demonstration of the network topology[33]. Enriched gene sets and linked genes were visualised using the Cytoscape.[34]

#### Accession codes

The list of the stringent CNVs detected in this study was submitted to the dbVar database at NCBI (https://www.ncbi.nlm.nih.gov/dbvar) under accession number **nstd157**.

## Results

### Samples and CNV descriptive statistics

**Fig 2:**
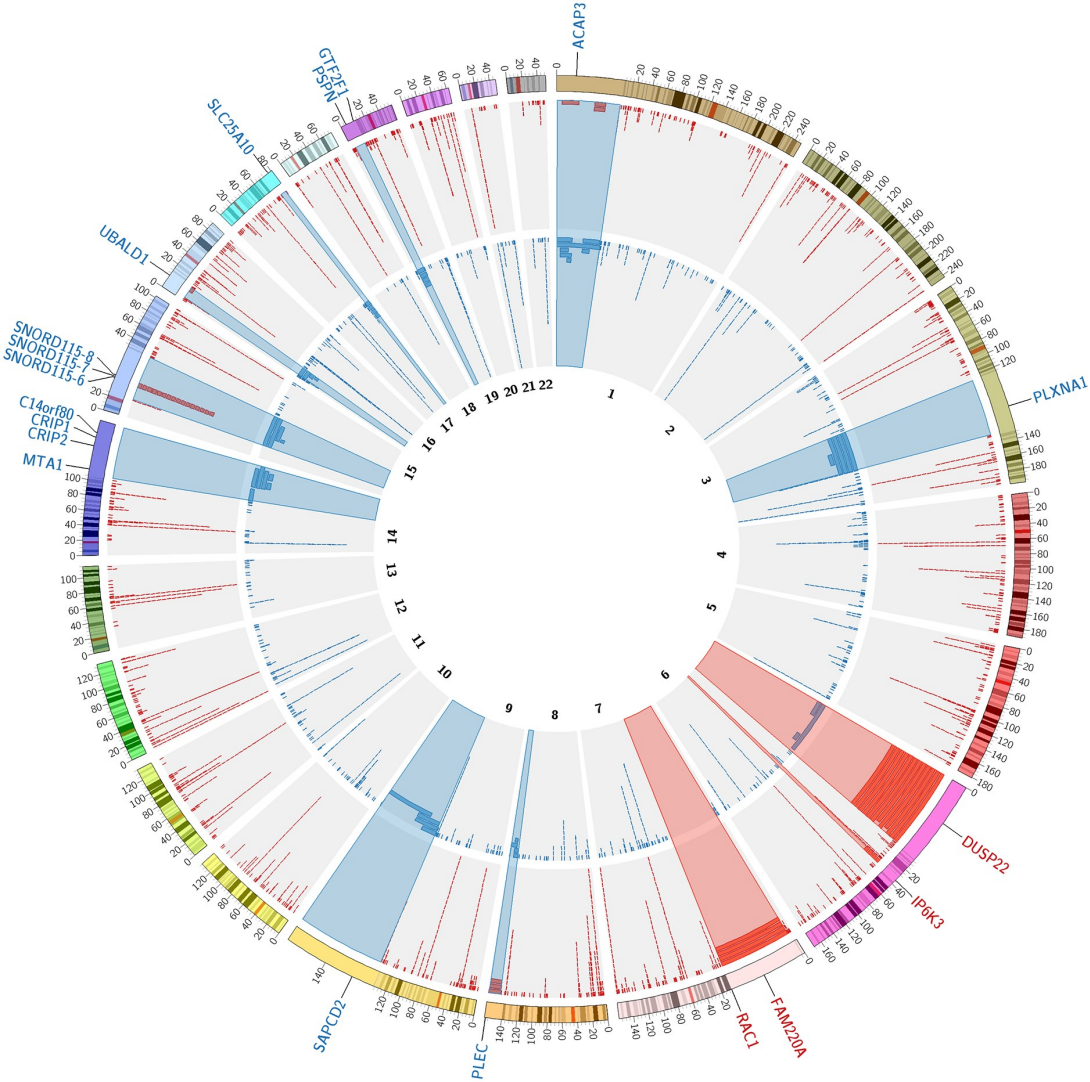
Chromosome view of detected CNVs. The CNVs are presented as tiles on the corresponding genomic positions. The colour of the tile indicates the CNV type: deletions are red, duplications are blue. The height of tile stack in each genomic region corresponds to the number of CNVs; if genomic region contains more than 25 CNVs, only 25 tiles are presented. The genes intersected by rare CNVs and significantly (P_adj_<0.01) associated with IBD are marked by red (for deleted genes) or blue (for duplicated genes) labels; the colocated genes overlapped by the same CNVs with no significant IBD association are not presented. The regions contained the genes intersected by rare CNVs significantly associated with IBD are zoomed in 1000 times and highlighted (red and blue highlights for the associated deletions and duplications, correspondingly). The figure was built using the Circos[35] tool.

During the quality control procedures, a total of 26 out of the 269 samples were removed from further analysis (see Fig 1). Of those, five samples were excluded due to insufficient call rate and/or exceeded SD of LRR and BAF related to poor sample quality and genotyping errors. Another 18 samples were removed for sample relatedness, population outliers or inconsistency between self-reported sex. Three samples were disqualified after CNVs calling and merging due to an exceeded number of detected CNVs. CNVs identified in the remaining 243 IBD samples were included in the study. Of these samples, 120 were from patients with CD (51 males and 69 females) and 123 were from patients with UC (55 males and 68 females). After CNVs quality control and merging the results of three CNV calling algorithms, 4,402 stringent CNVs were kept for the further analysis, which included 2,872 deletions and 1,530 duplications (Fig 2). The overall number of CNVs and number of short (<100 kbp) CNVs in CD samples was significantly higher than that in UC samples (Table 1). Overall, 1,984 CNVs (45%) overlapped at least one gene (genic CNVs). Of those, 1,018 and 966 were found in CD and UC samples, respectively. The majority of detected CNVs (88%) were less than 100 kbp in size. However, 205 samples (84%) contained at least one CNV with size larger than 100 kbp. 33 samples (13%) contained CNVs longer than 500 kbp and 13 samples (5%) had CNV covering more than 1 Mbp (Million base pair). Of those, seven very large CNVs were observed in CD samples, and six such CNVs were detected in UC samples, including five and four genic CNVs, correspondingly. The number of short (<100 kbp) deletions observed in CD cases significantly exceeded their number found in UC cases (Fisher’s exact test p-value < 0.05). More no-genic CNVs and especially no-genic deletions were observed in CD than in UC. The numbers of genic CNVs in CD and UC samples were not significantly different in all sizes and type groups (see Table 1).

**Table 1:** All CNVs, genic CNVs and no genic CNVs of different lengths observed in the samples from CD and UC populations. Summary data for all CNV types and separated data for deletions and duplications are provided.

CNV Length^1^	All				Genic				No-genic			
	IBD	CD	UC	P^2^	IBD	CD	UC	P^2^	IBD	CD	UC	P^2^
**All types**												
Any Length	4402	2297	2105	**0.008**	1984	1018	966	0.220	2418	1279	1139	**0.014**
5–100 kbp	3879	2028	1851	**0.010**	1622	838	784	0.198	2257	1190	1067	**0.025**
100–500 kbp	477	247	230	0.481	336	167	169	0.939	141	80	61	0.237
500 kbp—1 Mbp	33	15	18	0.808	17	8	9	1	16	7	9	1
>1 Mbp	13	7	6	1	9	5	4	1	4	2	2	1
**Deletions**												
Any Length	2872	1497	1375	**0.036**	1012	518	494	0.428	1860	979	881	**0.047**
5–100 kbp	2590	1353	1237	**0.039**	841	437	404	0.288	1749	916	833	0.076
100–500 kbp	253	130	123	0.661	162	76	86	0.661	91	54	37	0.188
500 kbp—1 Mbp	20	9	11	1	4	2	2	1	16	7	9	1
>1 Mbp	9	5	4	1	5	3	2	1	4	2	2	1
**Duplications**												
Any Length	1530	800	730	0.115	972	500	472	0.369	558	300	258	0.154
5–100 kbp	1289	675	614	0.128	781	401	380	0.452	508	274	234	0.153
100–500 kbp	224	117	107	0.575	174	91	83	0.597	50	26	24	0.846
500 kbp—1 Mbp	13	6	7	1	13	6	7	1	0	0	0	n/a
>1 Mbp	4	2	2	1	4	2	2	1	0	0	0	n/a

^1^ kbp: kilobase pair; Mbp: Million base pair;

^2^ For each CNV type and length, Fisher’s exact test was applied to compare observed and expected numbers of CNVs detected in CD and UC samples;

p-values are provided, significant associations are **bolded**.

### CNV affected genes

Overall, 7,061 genes were intersected by at least one CNV (deletion or duplication) in at least one analyzed sample; 4,347 and 4,416 genes were overlapped by deletions and duplications, correspondingly. For each gene, we compared the number of overlapping CNVs in IBD population with the number of overlapping CNVs in control population (see Fig 1). This comparison was conducted separately for deletions and duplications. After the correction of p-values for multiple testing over all 8,763 comparisons, 162 CNV-overlapped genes and pseudogenes passed the p-value significance threshold (P_adj_) of 0.01. Most of these genes were overlapped by CNVs in more than 0.1% of analyzed control samples (i.e. by common CNVs). Of 116 genes overlapped by deletions, four genes were covered by three CNVs observed in less than 0.1% of control samples, which could be considered as rare IBD-associated deletions. Of 56 genes overlapped by duplications, fourteen genes were overlapped by nine rare IBD-associated duplications (Table 2). The list of gene sets linked to genes, overlapped by rare IBD-associated CNVs is presented in the S1 Table.

**Table 2:** The genes affected by rare CNVs overrepresented in IBD population.

locus	symbol	case (CD/UC)	Control	OR (95% CI)^2^	P_adj_^3^	CNVs length (kbp)
**Deletions**						
6p25.3	DUSP22	12 (4/8)	1	154.6 (22.7–6349.5)	8.8×10^−11^	108.4–113.6
6p21.31	IP6K3	4 (1/3)	0	Inf (8.2-Inf)	2.7×10^−03^	5.7
7p22.1	FAM220A, RAC1	4 (2/2)	0	Inf (8.2-Inf)	2.7×10^−03^	100.4
**Duplications**						
1p36.33	ACAP3	5 (3/2)	2	31.3 (5.1–327.6)	7.2×10^−03^	9.0–87.0
3q21.3	PLXNA1	5 (3/2)	2	31.3 (5.1–327.6)	7.2×10^−03^	23.2–78.4
8q24.3	PLEC	4 (3/1)	0	Inf (8.2-Inf)	6.0×10^−03^	5.9–11.9
9q34.3	SAPCD2	4 (3/1)	0	Inf (8.2-Inf)	6.0×10^−03^	29.6–100.0
14q32.33	MTA1, CRIP2, CRIP1, C14orf80	5 (2/3)	0	Inf (11.4-Inf)	9.2×10^−04^	13.9–44.7
15q11.2	SNORD115-6, SNORD115-7, SNORD115-8^1^	4 (1/3)	0	Inf (8.2-Inf)	6.0×10^−03^	6.0–62.2
16p13.3	UBALD1	4 (2/2)	0	Inf (8.2-Inf)	6.0×10^−03^	6.0–16.2
17q25.3	SLC25A10	4 (2/2)	0	Inf (8.2-Inf)	6.0×10^−03^	6.6–7.6
19p13.3	PSPN, GTF2F1	4 (2/2)	0	Inf (8.2-Inf)	6.0×10^−03^	12.0–13.2

^1^RNA genes;

^2^OR: odds ratio and CI: confidence interval;

^3^P_adj_: adjusted P-value

#### Deletions

Twelve IBD samples demonstrated relatively long (109–114 kbp) deletions located in the 6p25.3 cytoband and intersected the *DUSP22* gene. This deletion was observed only in one control sample and was considered as rare deletion significantly associated with IBD (Odds ratio (OR) = 154.6, 95% Confidence Interval (CI) = 22.7–6349.5, P_adj_ = 8.8×10^−11^). According to Gene Ontology (GO) database, the functions of the *DUSP22* include the JNK signalling pathway activation, positive regulation of JUN kinase activity and MAPK inactivation.

Another short deletion on the chromosome 6 was observed in the p21.31 locus in four IBD samples with no corresponding CNV in control samples (P_adj_ = 2.7×10^−3^). The deletion overlapped the *IP6K3* gene, which is functionally involved in inositol phosphate metabolism.

The third deletion (7p22.1 cytoband) significantly associated with IBD in this study was found in four IBD samples. No corresponding CNV was observed in control samples (P_adj_ = 2.7×10^−3^). The deletion intersected two genes: the *RAC1* and the *FAM220A*. The *RAC1* gene is included in numerous functional gene sets (GO terms, KEGG and Reactome pathways) associated with immune response and inflammation. The *FAM220A* gene also known as STAT3-interacting protein as a repressor (*SIPAR*) is implicated in the regulation of STAT-signaling pathway.[36]

#### Duplications

Five IBD samples carried duplications in the 1p36.33 locus overlapping the *ACAP3* gene, which is associated with endocytosis accordingly to KEGG pathways. The observed duplications differed in sizes from 9kbp to 87kbp. The longest duplication was detected in a male UC patient and spanned over eight genes (*SCNN1D*, *ACAP3*, *PUSL1*, *CPSF3L*, *GLTPD1*, *TAS1R3*, *DVL1*, and *MXRA8*). Three other samples had the duplication overlapping the *SCNN1D* and *ACAP3* genes; and one sample had a short duplication over the *ACAP3* only. The *ACAP3* gene was overlapped by duplications in two control samples and thus was significantly associated with IBD (OR = 31.3, CI = 5.1–327.6, P_adj_ = 7.2×10^−3^). The *SCNN1D* genes did not pass the significance threshold after the FDR-correction. Other genes, overlapped by long duplications in one IBD sample with corresponding duplication in one control sample, were not significantly associated with IBD (number of samples with CNVs, OR and P_adj_ are presented in the S2 Table).

The *PLXNA1* gene located in the 3q21.3 cytoband was intersected by duplication in five IBD and two control samples (OR = 31.3, CI = 5.1–327.6, P_adj_ = 7.2×10^−3^). Four samples carried 78kbp long duplication, completely overlapping the *PLXNA1* gene, and one sample had a shorter CNV (23kbp) partially overlapped the gene. The *PLXNA1* gene is involved in the axon guidance through the semaphorin receptor activity.

Four IBD samples demonstrated various size duplications in the 8q24.3 cytoband, partially overlapping the *PLEC* gene. Due to the absence of corresponding CNVs in the control population, this duplication was significantly associated with IBD (P_adj_ = 6.0×10^−3^). The *PLEC* gene encodes Plectin, one of the cytolinker proteins, which plays an important role in maintaining cell and tissue integrity, and also participates in assembly and regulation of signaling complexes.[37]

Duplications of different sizes (30-100kbp) were found in the 9q32.33 cytoband in four IBD samples. The longest duplication spanned over six genes (*SAPCD2*, *UAP1L1*, *MAN1B1*-*AS1*, *MAN1B1*, *DPP7*, and *GRIN1*). Two IBD patients had the duplications partially overlapping the first one and spanned over the *NPDC1*, *ENTPD2*, *SAPCD2*, *UAP1L1*, *MAN1B1-AS1* and *MAN1B1* genes and another sample had a short duplication over the *NPDC1*, *ENTPD2* and *SAPCD2* genes. No corresponding CNVs were found in the control samples, however, only the *SAPCD2* was significantly associated with IBD (P_adj_ = 6.0×10^−3^), while genes overlapped by only three CNVs did not pass the significance threshold (P_adj_ = 0.035). *SAPCD2* plays a role in planar mitotic spindle orientation during the asymmetric cell divisions in epithelium and retina morphogenesis.[38]

Six duplications with different breakpoints were observed in 14q32.33 with no corresponding CNVs in control samples. Three samples with CNVs spanned over four genes (*MTA1*, *CRIP2*, *CRIP1* and *C14orf80*); one CD sample had CNV affected only *MTA1* and *CRIP2* and one UC sample had two duplications: one covered the MTA1 gene and another overlapped the *CRIP1* and *C14orf80* genes. *MTA1* and *CRIP2* are functionally related to the NF-κB protein complex.[39,40] The *CRIP1* gene presumably has a role in the intestinal zinc absorption and intracellular zinc transport.[41] The porcine *CRIP1* orthologous is associated with the gut immunity.[42] The functions of the *C14orf80* gene are unknown.

Two IBD samples had relatively long (62kbp) duplication spanned over 30 RNA genes: *PWAR4* and 29 *SNORD115* genes encoding the small nucleolar RNA in 15q11.2 region (C/D Box 115 Cluster). This CNV was partially overlapped by 31kbp long duplication covered 17 genes from the same cluster and by short (6kbp) duplication covered only the *SNORD115-6*, *SNORD115-7* and *SNORD115-8* in two other IBD samples. Due to the absence of corresponding duplications in control samples the *SNORD115-6*, *SNORD115-7* and *SNORD115-8* genes were significantly associated with IBD (P_adj_ = 6.0×10^−3^). It has been reported that the C/D box snoRNA can affect the alternative spicing. In particular, the neuron-specific *SNORD115* alternates the exon selection of the serotonin receptor 2C pre-mRNA.[43]

Four IBD samples carried duplications intersecting the *UBALD1* gene in the 16p13.3 locus. Two of these samples had longer CNVs spanned also over C16orf96 gene. The *UBALD1* gene was not overlapped by any CNV in control samples and passed the significance threshold with P_adj_ = 6.0×10^−3^. The functions of the *UBALD1* gene is yet unknown, however, it was associated with IL-8 secretion and NF-kappa-B signalling.[44]

Short (6.6–7.6kbp) duplications in the 17q25.3 cytoband partially overlapped the *SLC25A10* in four samples from the IBD population. No corresponding duplications were observed in control samples (P_adj_ = 6.0×10^−3^). The *SLC25A10* gene is functionally involved in the metabolism as transmembrane transporter.

Finally, 12-13kbp long duplications in the 19p13.3 cytoband were found in four IBD samples with no corresponding CNVs in control population (P_adj_ = 6.0×10^−3^). The duplication spanned over the *PSPN* and *GTF2F1* genes; longer CNVs in two samples covered also the *ALKBH7* gene, which was not associated with IBD (P_adj_ = 0.27). The *PSPN* gene is functionally linked to the MAPK activity, while *GTF2F1* is related to DNA and RNA binding.

### Association analysis of rare CNVs and IBD clinical characteristics

Overall, the above-mentioned genes were covered by CNVs in 40 (17 CD and 23 UC) out of the 243 IBD samples. Of these, 10 CD and 19 UC samples had only one of the described regions, and five CD and four UC samples contained up to six different CNVs significantly associated with IBD in the current study (S3 Table). We assessed the association of clinical characteristics of the IBD patients with these rare CNVs, and did not find any significant difference in the rare CNV distribution between groups of CD and UC patients, as well as groups of IBD patients with different ages at onset, CD and UC phenotypes including CD and UC process location and CD behavior, and IBD psychiatric comorbidity (S4 Table).

### Non-genic CNVs

Overall, 2,418 (1,279 in CD and 1,139 in UC samples) CNVs detected in IBD samples did not overlap any gene. The overall numbers of no genic CNVs, particularly very short no-genic CNVs and very short no genic deletions observed in CD cases were significantly larger than these numbers in UC cases (p-values = 0.014, 0.025 and 0.047 correspondingly).

### Gene set overrepresentation analysis

In general, genes overlapped by rare IBD-associated CNVs were included in 1,016 gene sets from four annotation gene set libraries (S1 Fig, S1 Table). Large group of these gene sets are functionally related to such processes as regulation of transcription, RNA and DNA binding and DNA repair (denoted as “DNA and RNA processes”), and also to the regulation of gene expression, including epigenetic regulation by histone modifications. 79 gene sets are directly related to the regulation of acute and chronic immune-inflammatory response, including numerous relevant signalling pathways, cell migration and cytotoxicity. Other large groups of gene sets are related to such processes as tissue development and morphogenesis, axon guidance and signal transduction pathways.

We tested the set of CNVs affected genes for the overrepresentation in the functional gene sets to identify potentially involved biological processes. 61 genes associated with IBD with a P_adj_<0.05 were included in gene set enrichment analysis. There are only three gene sets overlapped the IBD-associated gene list by at least two genes and were enriched in genes affected by rare IBD-associated CNVs with the adjusted p-value <0.05. These included two Reactome pathways linked to *RAC1* and *PLXNA1* (“Sema3A PAK dependent Axon repulsion” and “SEMA3A-Plexin repulsion signaling by inhibiting Integrin adhesion”) and one GO term “nucleotide-sugar biosynthetic process” included two genes with low significance of the IBD-association (*GMDS* and *UAP1L1*).

## Discussion

In the current study, we investigated rare genic CNVs detected by genome-wide high-resolution microarray technology in a cohort of 243 IBD patients and 2,988 control samples. Of all CNVs identified simultaneously by at least two computational algorithms, 65% were deletions. This imbalance is most likely related to the detection bias of SNP-based array platforms leading to the missing of duplications.[9,28] 35% of the deletions overlapped one or more genes, while the proportion of gene-overlapping duplications reached 64%. This difference can be explained by lower negative selective pressure on duplications than on deletions because of their milder phenotypic effect.[8] About 88% of the discovered CNVs were less than 100kbp in size. Although the burden of short CNVs, especially short deletions, was significantly higher in the group of samples with CD than with UC, such difference was not observed in the burden of all CNVs in CD and UC (see Table 1), suggesting that overall CNV burden did not differ significantly between IBD subtypes. Although we detected the CNVs more frequent in controls compared with the IBD patients, the overall CNV burden did not significantly differ in the IBD patients compared with the controls in all of the CNV length types, except the largest CNV length type (>1 Mbp), where the burden of the large CNVs in the IBD patients is significantly higher than that in the controls (p-value = 0.002, OR = 2.8 and 95% CI: 1.4–5.2).

The results of gene-based analysis outlined several rare genic CNVs significantly overrepresented in IBD population, which may have an important role in the IBD pathogenesis. We found eighteen protein-coding genes intersected by rare CNVs significantly associated with IBD. Seven of these genes (*CRIP1*, *DUSP22*, *GTF2F1*, *IP6K3*, *MTA1*, *PSPN* and *RAC1*) were included in the functional gene sets related to the development of inflammation directly or by performing the regulatory functions in the signalling pathways. Three other genes (*CRIP2*, *FAM220A* and *UBALD1*) were linked to the immune-inflammatory process by previous studies. [36,40,44] A large part of the genes was involved in the activity of signal transduction pathways, such as MAPK (*DUSP22*, *IP6K3* and *PSPN*) and NF-κB protein complex (*MTA1*, *CRIP2*, *DUSP22*, *UBALD1*, *SAPCD2* and *SLC25A10*). Of particular interest were the duplicated genes *UBALD1* (previously known as *FAM100A*), *SAPCD2* (also known as *C9orf140*) and *SLC25A10*, which were associated with the regulation of IL-8 secretion and NF-kappa-B signalling in the study of NOD2 functions related to CD.[44] As one of the small regulatory GTPases with pro-inflammatory effect, *RAC1* influences the MAPK activity as well as the NF-κB signalling cascades, regulates the neutrophil functions, and also has a stimulating effect on the NADPH oxidase activity in macrophages. Recent studies showed the association of the increased activity of *RAC1* with an inflammatory response in IBD [45]. In turn, the *GTF2F1* gene demonstrated upregulation in response to enterovirus infection.[46]

The highly significant IBD associated deletion over the *DUSP22* gene deserves a special interest. The *DUSP22*, also known as *JKAP* (JNK pathway-associated phosphatase), inhibits T-cell proliferation and cytokine production by JNK activation.[47] In mouse model, the *DUSP22* suppressed T-cell immune responses and the development of autoimmunity.[47] It was also found in human study, that downregulation of *DUSP22* in T cells was associated with the disease activity in the individuals with systemic lupus erythematosus.[48]

Interestingly, previous GWAS studies have identified genetic variants near the *DUSP22* gene associated with IBD[3] and celiac disease[49]. In addition, two SNPs were linked to macrophage inflammatory protein 1α level[50] and the neutrophilic and eosinophilic blood indices, which in turn were associated with such immune-mediated diseases as asthma, rheumatoid arthritis, celiac disease and type I diabetes, but not with IBD[51] (S2 Fig). Here, our findings provide the additional support for putative role of *DUSP22* in the development of chronic intestinal inflammation. Accordingly to GWAS, the *IP6K3* gene was reported as a candidate gene for the association with CD.[52] In addition, the locus of *ACAP3* gene was first linked to IBD in[6] and then reviewed [4]. Other genes were previously associated with such traits as immunoglobulin G N-glycosylation, body mass index and cholesterol level, and such diseases as asthma, coronary artery disease and dental caries (S5 Table).

The functional gene set overrepresentation analysis revealed three significant pathways potentially involved in the risk of the disease. Interestingly, these pathways linked with brain related genes, such as *PLXNA1*; *RAC1*. Previous studies showed that de novo missense variants in the RAC1 gene associated with individuals presenting with intellectual disability and brain malformations. [49] Numerous epidemiological studies have reported a high frequency of psychiatric disorders, especially mood disorders in persons with IBD. [50] These results suggest genetic factors may play a potential role in the development of psychiatric comorbidity in IBD. Furthermore, we also observed some other immune process related gene sets were marginally significant in the genes affected by rare IBD-associated CNVs with the adjusted p-value of the overrepresentation < 0.25 (S3 Fig). Of these gene sets overlapped the IBD-associated gene list by at least two genes, six were directly linked to the immune system (Reactome pathways: “HIV Infection”, “Infectious disease”, “FCERI mediated MAPK activation”, “Fc epsilon receptor (FCERI) signaling”, “DAP12 signaling” and “DAP12 interactions”). Other large groups of gene sets are involved in intracellular signalling pathways and diverse regulatory, metabolic and developmental functions.

There are some limitations in this study. There was a lack of validation of the top CNVs identified in this study. In addition, the association of these top CNVs with the risk of IBD did not demonstrate any causality. We will collaborate with other research groups to further validate these findings. The CNVs from the control group were generated in other studies. Although we made efforts to ensure that the analysis was focused on the individuals with Caucasian ethnicity in both the control and IBD groups and the microarrays used to generate the genotype data were similar, it was likely that there were some other potential confounding factors we did not control in the analysis, which may bias the results.

In summary, our research revealed additional loci to the genetic susceptibility of IBD. The IBD-associated genes we identified, which are not seemingly related to the immune or inflammatory processes, conduct essential functions in cell metabolism that can indirectly impact the inflammatory process. Similar to previous genome-wide association studies that have highlighted the presence of genes common to both UC and CD, and shared with some other autoimmune disorders, some of the rare CNV regions we identified are also shared by both UC and CD. For example, the deletion region 6p25.3 is shared by 8 UC patients and 4 CD patients, respectively. This further suggests that the etiology of UC and CD may involve with a similar genetic component.

## Supporting information

S1 Fig Visualization of the gene sets contained genes, overlapped by rare IBD-associated CNVs. Circle nodes represent genes (overlapped by deletion, red; overlapped by duplication, blue). Other nodes represent gene sets from four gene set libraries; gene sets contain more than one gene overlapped by rare IBD-associated CNV marked by pink colour. Functionally related gene sets are grouped and labelled. Edges connect gene sets and CNV-overlapped genes; colour represents the type of CNV (deletion, red; duplication, blue).
(PDF)

S2 Fig Visualization of the cytogenetic bands, genomic coordinates, genes and rare CNVs from UCSC browser (genome.ucsc.edu) with GRCh37/hg19 genome assembly. Red bars represent deletions; blue bars represent duplications. Genetic variants, presented in the NHGRI-EBI Catalog of published genome-wide association studies (GWAS Catalog) are highlighted: 1) *rs55713716* is associated with eosinophil counts, eosinophil percentage of granulocytes, eosinophil percentage of white cells, neutrophil percentage of granulocytes and sum eosinophil basophil counts (Astle 2016, Pubmed ID: 27863252); 2) *rs6900267* variant is associated with macrophage inflammatory protein 1α level (Ahola-Olli 2016, Pubmed ID: 27989323); 3) *rs7773324* is associated with Crohn’s disease and IBD (Liu 2015, Pubmed ID: 26192919); and 4) *rs1033180* is associated with celiac disease (Dubois 2010, Pubmed ID: 20190752).
(PDF)

S3 Fig A gene set overrepresentation map. Gene set overrepresentation analysis results were mapped as a network of gene sets (nodes shape corresponds to gene set library), related to the corresponding genes associated with IBD in the current study (circle nodes). The colour of the gene node indicates the type of CNV (deletion, red; duplication, blue) overlapped the gene in the current study. The colour of the gene set node corresponds to the p-value adjusted using the Benjamini-Hochberg method for correction for multiple hypotheses testing of gene set enrichment. The IBD-associated genes not implicated in the enriched gene sets are not shown. The edges represent the implication of genes in the enriched gene sets.
(TIF)

S1 Table The list of gene sets (GO terms, KEGG and Reactome pathways) contain the genes, intersected by the rare IBD-associated CNVs. Of GO terms, only “GO biological process” and “GO molecular function” domains are provided. Each gene sets included in the group of functionally related gene sets.
(XLSX)

S2 Table List of genes overlapped by the same CNVs with the genes significantly associated with IBD. IBD-associated and collocated genes are presented, IBD-associated genes are marked bold.
(XLSX)

S3 Table Clinical and genetic features of patients with CNV-overlapped genes associated with IBD in current study. (XLSX)

S4 Table The distribution of clinical features among samples with rare CNV regions overrepresented in IBD population. (XLSX)

S5 Table The list of GWAS catalogue entries related to genes associated with IBD in the current study. Entries corresponded to IBD, CD or UC are highlighted, corresponding genes are marked bold.
(XLSX)
