# Supplementary figures and images for: Genome-wide analysis identifies rare copy number variations associated with inflammatory bowel disease
Source: PLoS One. 2019 Jun 11;14(6):e0217846. doi: 10.1371/journal.pone.0217846 (PMC6559655; doi:10.1371/journal.pone.0217846)

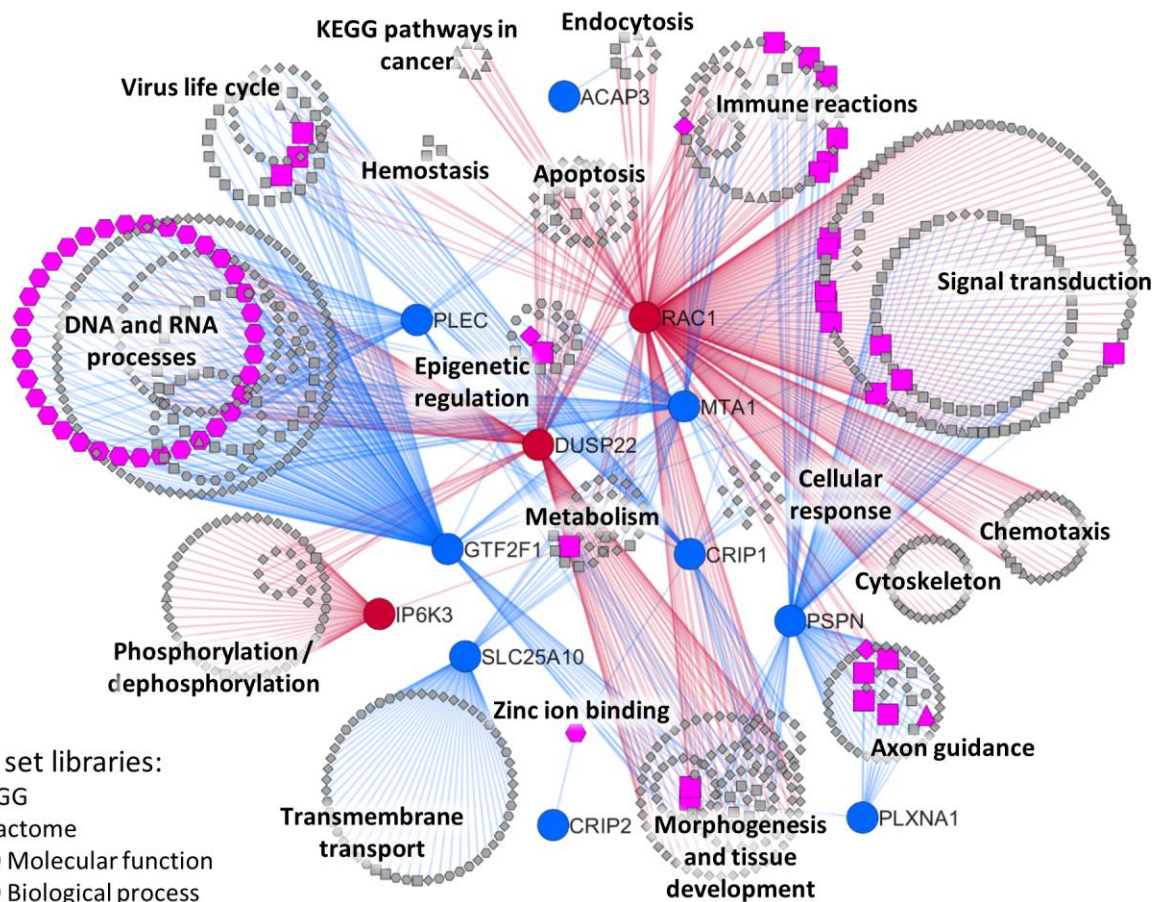

Supplement: S1 Fig — Circle nodes represent genes (overlapped by deletion, red; overlapped by duplication, blue). Other nodes represent gene sets from four gene set libraries; gene sets contain more than one gene overlapped by rare IBD-associated CNV marked by pink colour. Functionally related gene sets are grouped and labelled. Edges connect gene sets and CNV-overlapped genes; colour represents the type of CNV (deletion, red; duplication, blue). (PDF) [file pone.0217846.s001.pdf]

chr6 (p25.3)

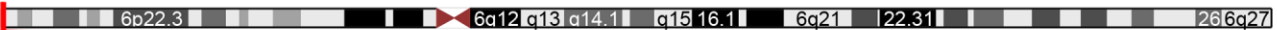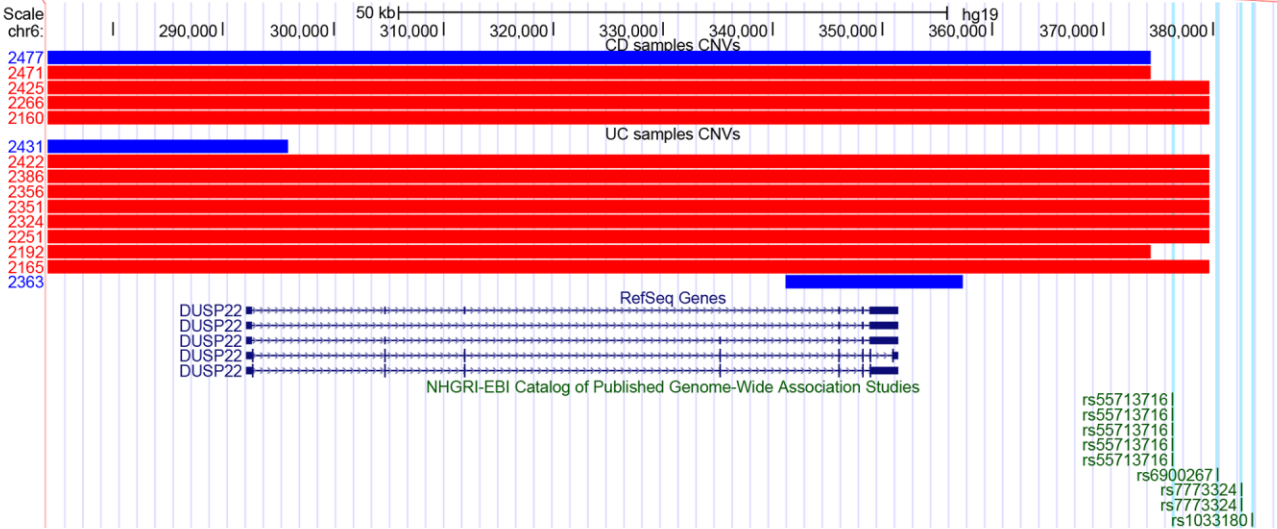

Supplement: S2 Fig — Red bars represent deletions; blue bars represent duplications. Genetic variants, presented in the NHGRI-EBI Catalog of published genome-wide association studies (GWAS Catalog) are highlighted: 1) rs55713716 is associated with eosinophil counts, eosinophil percentage of granulocytes, eosinophil percentage of white cells, neutrophil percentage of granulocytes and sum eosinophil basophil counts (Astle 2016, Pubmed ID: 27863252); 2) rs6900267 variant is associated with macrophage inflammatory protein 1α level (Ahola-Olli 2016, Pubmed ID: 27989323); 3) rs7773324 is associated with Crohn’s disease and IBD (Liu 2015, Pubmed ID: 26192919); and 4) rs1033180 is associated with celiac disease (Dubois 2010, Pubmed ID: 20190752). (PDF) [file pone.0217846.s002.pdf]

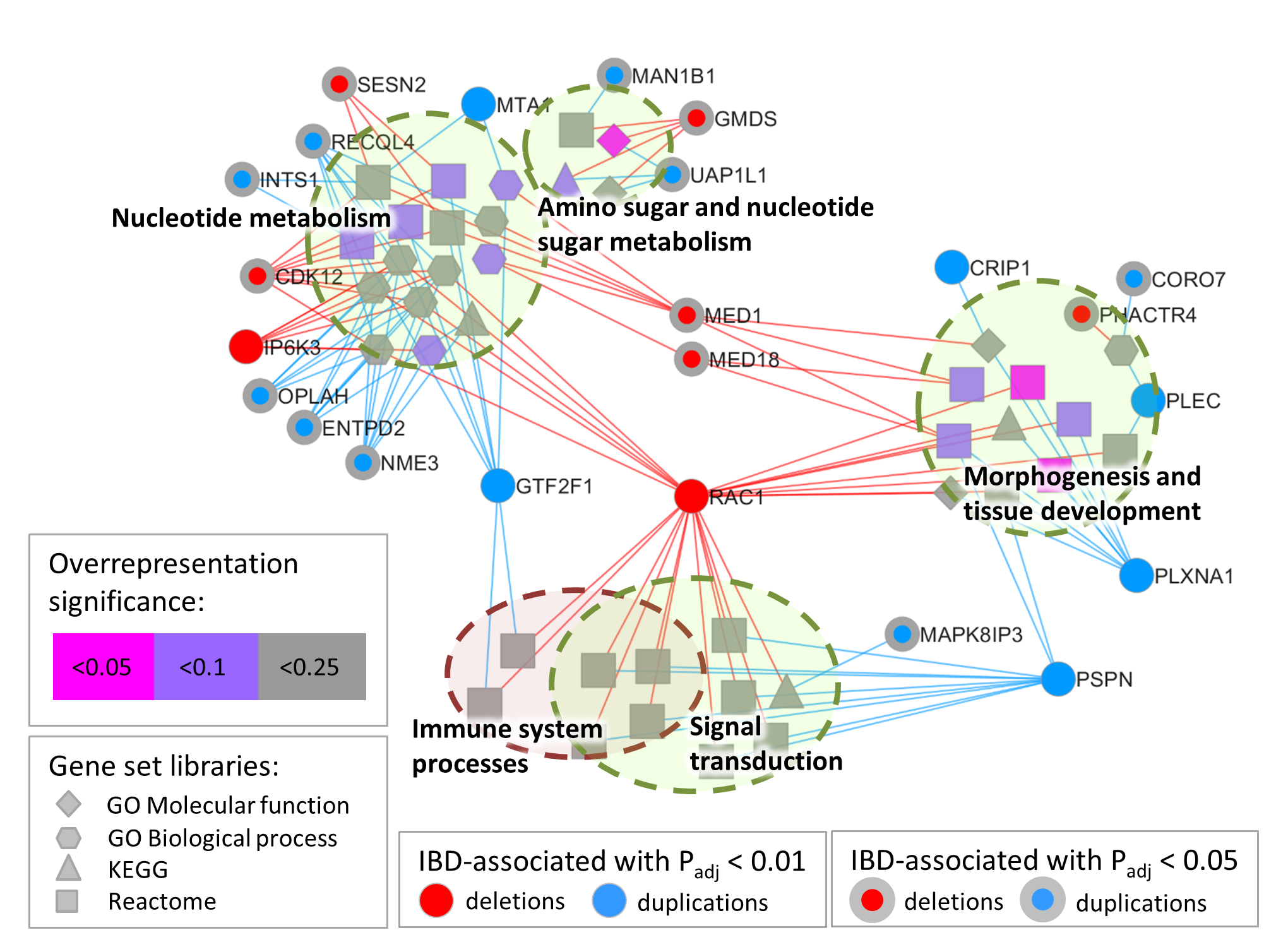

Supplement: S3 Fig — Gene set overrepresentation analysis results were mapped as a network of gene sets (nodes shape corresponds to gene set library), related to the corresponding genes associated with IBD in the current study (circle nodes). The colour of the gene node indicates the type of CNV (deletion, red; duplication, blue) overlapped the gene in the current study. The colour of the gene set node corresponds to the p-value adjusted using the Benjamini-Hochberg method for correction for multiple hypotheses testing of gene set enrichment. The IBD-associated genes not implicated in the enriched gene sets are not shown. The edges represent the implication of genes in the enriched gene sets. (TIF) [file pone.0217846.s003.tif]
